# Pilot clinical study of Taxifolin Aqua effect in patients with amnestic mild cognitive impairment

**DOI:** 10.3389/fpsyt.2026.1814042

**Published:** 2026-09-28

**Authors:** Elena V. Ponomareva, Natalya D. Selezneva, Irina S. Boksha, Olga K. Savushkina, Tatyana A. Prokhorova, Elena B. Tereshkina, Svetlana A. Zozulya, Svetlana I. Gavrilova, Igor V. Kolykhalov

**Affiliations:** Federal State Budgetary Scientific Institution Russian Mental Health Research Centre (FSBSI RMHRC), Moscow, Russia

**Keywords:** antio×idant, biochemical blood analysis, mild cognitive impairment, Taxifolin, treatment response

## Abstract

Taxifolin (dihydroquercetin), a potent antioxidant flavonoid, shows promise for neurodegenerative conditions. Our pilot study aimed at evaluation the efficacy and safety of Taxifolin in patients with amnestic mild cognitive impairment (aMCI) and monitored a set of blood measures. Twenty-three patients (18 female; aged 53-87) received dihydroquercetin oral solution for 8 weeks with a 4-week follow-up period in an open-label study design. Thirteen patients with aMCI were monitored as controls. Outcomes were assessed using cognitive status (MMSE, MoCA, FCSRT-IR), neuropsychiatric symptoms’ (NPI-Q, HDRS), and rater's impression (CGI) scales, along with blood measures monitoring (glutathione reductase (GR), glutamate dehydrogenase (GDH), and superoxide dismutase (SOD); plasma neutrophil elastase (NE), alpha-1-proteinase inhibitor (alpha-1PI)). Responder classification using composite Z-score changes was based on all clinical scales. Results: Eleven patients were classified as responders, showing significant MMSE and MoCA improvement (mean +2.73 points, p=0.001) sustained through 4-week follow-up. Non-responders and control group showed slight cognitive decline. Composite Z-score changes at the 4-week follow-up revealed differences between responders/non-responders (ANOVA F(2,20)=17.38, p=0.001); biomarker analysis revealed difference in NE/alpha-1PI ratio (p=0.034), SOD (p=0.023), and GR dynamics (p=0.022), which correlated negatively with clinical response (R=-0.547, p=0.007), and baseline GDH (p=0.049). Conclusion: Taxifolin treatment resulted in significant cognitive benefits in ~half of aMCI patients, with effects sustained for one month post-treatment. Biomarker changes reveal possible mechanisms involving oxidative stress modulation and inflammatory pathways. These findings support further controlled trials of Taxifolin in populations at risk of Alzheimer's disease.

## Background

Taxifolin, a flavonoid known as dihydroquercetin (3,5,7,3,4-pentahydroxy flavanone), exhibits pharmacological properties akin to other flavonoids. However, it boasts a stronger antioxidant capability. Found in various plants, taxifolin is plentiful, and its medicinal potential is increasingly appreciated (1–3). Aim. The pilot clinical study sought to evaluate the effectiveness and safety of a Taxifolin Aqua treatment regimen—an aqueous solution of dihydroquercetin—for patients with mild cognitive impairment of the amnestic type (aMCI). The goal was to determine its potential as a therapy for cognitive and neuropsychiatric issues in individuals at risk for Alzheimer's disease (AD). Additionally, the study aimed to bolster research by monitoring blood biomarkers. Methods. The study had a prospective, open-label design; it was approved by the Ethical Committee of the MHRC Protocol No 922 from 19.02.2024, and it adheres to principles of the Helsinki Declaration of the World Medical Association, as amended in 1975-2024. The study excluded participants with the following conditions: a diagnosis of dementia according to ICD-10 criteria, neurological disorders such as metabolic encephalopathies, Parkinson’s disease, stroke, epilepsy, infectious diseases, demyelinating or hereditary degenerative diseases of the central nervous system, brain tumors, traumatic brain injuries, systemic metabolic diseases, mental disorders, severe organ pathology including renal and hepatic failure, malignant extracerebral tumors, HIV infection, uncontrolled diabetes, or other endocrine disorders. It also excluded individuals with alcohol or drug dependence, current intoxication, signs of exacerbated allergic, inflammatory, or autoimmune diseases, recent vaccination, an HDRS score greater than 18, or systolic and diastolic blood pressure exceeding 180 and 95 mmHg, respectively. Additionally, participants using immunotropic drugs within the last three months were not eligible.

## Patients

The study enrolled 23 patients (18 postmenopausal women and 5 men aged 53 to 87 years, all met aMCI criteria (4), with MMSE scores from 26 to 28, CDR of 0.5, and MHIS ≤4, complaints of mild memory loss confirmed by an informant (usually a family member), and objectively detectable signs of mild cognitive dysfunction, at least in tests of memory and those cognitive areas that are usually clearly impaired in AD. Their routine remained largely unchanged, although some intricate and instrumental tasks might have grown more challenging in their daily and professional lives. The patients received Taxifolin Aqua, i.e., dihydroquercetin oral solution (3 mg/ml), at a daily dose of 30 mg dihydroquercetin per day for 8 weeks, followed by a 4-week observation period. Taxifolin Aqua is manufactured by Korolev Pharm LLC and has a certificate of state registration of products No. RU.77.99.11.003E.003036 dated July 24th, 2018. The product was produced on February 07th, 2024, and is in series 001. The control group consisted of 13 aMCI patients: 3 men and 10 postmenopausal women aged 48 to 78. These individuals received no treatment and were inspected by the same team of psychiatrists during the same period of observation as patients who were treated with Taxifolin Aqua. All participants, including those in the control group, signed informed consent form. The database for statistical processing contained no missing data. No outliers were observed in respect to clinical data. As for biochemical data, all measurements were processed using appropriate statistical methods, depending on their distribution: normal or non-parametric.

For a comprehensive assessment of the therapy effectiveness, a methodology for stratifying patients into responder/non-responder groups was used, based on a composite method using standardized changes (Z-scores, please see **Supplementary Material**) on key cognitive and neuropsychiatric scales, such as CDR SoB—The Clinical Dementia Rating Scale Sum of Boxes; MHIS—the Modified Hachinski Ischemic Scale; MMSE—the Mini Mental State Examination (5); HDRS (or HAM-D)—the Hamilton Depression Rating Scale (https://www.sciencedirect.com/topics/neuroscience/hamilton-rating-scale-for-depression); CGI—the Clinical Global Impression Scale; MoCA—the Montreal Cognitive Assessment scale; MBI-C—the Mild Behavioral Impairment Checklist (6); FCSRT-IR—the Free and Cued Selective Reminding Test—Immediate Recall (7); NPI-Q—the Neuropsychiatric Inventory Questionnaire (https://www.dementiaresearch.org.au/wp-content/uploads/2016/01/NPI-Q_cr.pdf).

To thoroughly evaluate treatment effectiveness, we stratified patients into subgroups responders/non-responders using a rigorous methodology. This approach combined two independent methods: classification via the CGI scale and a composite method based on standardized changes (Z-scores) in key cognitive and neuropsychiatric scales (please, see **Supplementary Table 1**). The results showed a strong agreement between these methods: the overall percentage of agreement was 95.7%, and the weighted Cohen’s kappa coefficient was κ = 0.955 (p < 0.001). This high level of agreement underscores the reliability and validity of our composite measure for assessing treatment response. Patients were considered responders if clinically significant improvement was observed continuously throughout the observation period (up to 15 weeks), which was characterized by a persistent, prolonged response to therapy.

The study protocol required recording of all adverse events (AEs) that occurred after the patient signed the informed consent and until the end of their participation in the study. An AE is defined as “any medically unfavorable event identified in a clinical trial subject after administration of a medicinal product, which may or may not be causally related to its use” (National Standard of the Russian Federation “Good Clinical Practice” GOST R 52379-2005, dated September 25, 2005). If any AEs occur, the physician investigator must make the necessary entries in the primary documentation and then complete the corresponding pages of the patient’s Individual Registration Form.

In addition, the treatment course with Taxifolin Aqua was evaluated for its impact on various biological markers in patients’ blood (8) (please see **Supplementary Figure 1**). The biochemical measures were monitored only in patients who were treated with Taxifolin Aqua. The blood samples were analyzed accordingly to the routine practice taken in the MHRC. Namely, the blood samples were taken in the morning, fasting, before the testing cognitive functioning in a patient who visits a psychiatrist. All the samples were anonymized and individual codes were assigned to every sample, and the laboratory personnel was blinded. The blood samples were sampled into appropriate vacutainers and immediately fractionated into the blood components, and the isolated blood components (platelets, red blood cells, and plasma) were aliquoted, frozen, and kept under refrigeration at −80 °C (plasma) or −20 °C (platelets, red blood cells) until analyses. All measurements were triplicated. The blood measures included enzymatic activity determination in neutrophil elastase, NE, and inhibitory activity of alpha1-proteinase inhibitor, alpha-1PI, in plasma; superoxide dismutase isozyme levels, SOD, were measured by ELISA in plasma; as for cytochrome *c*-oxidase, creatine phosphokinase, CPK, glutamate dehydrogenase, GDH, glutathione reductase, GR, and glutathione-S-transferase, the measures were their enzymatic activity levels in platelet extracts; glutathione peroxidase, GPx, activity was measured in extracts of red blood cells.

## Results

Of the total 23 patients treated with Taxifolin Aqua, 11 patients were assigned to responders, and 12 to non-responders. Comparative analysis of demographic and clinical characteristics revealed that the responders were significantly younger (*p* = 0.048) and had lower scores on the MHIS (*p* = 0.008) than non-responders (please, see **Supplementary Table 2**). No differences in MRI data were found between responders and non-responders.

At the end of the observation period, the responders showed the most notable improvement in cognitive function: their MoCA scores significantly rose by an average of 2.73 points (Friedman test, *p* = 0.001). A 2.73-point (mean) improvement on the Montreal Cognitive Assessment (MoCA) scale during monitoring of patient state is a clinically meaningful improvement in a person’s cognitive function. In contrast, non-responders experienced a decline in cognitive abilities, mirroring the trend seen in the control group (**Supplementary Figure 1**).

The effect of therapy on scores by the NPI, HDRS, and MBI-C was less pronounced and insignificant. Analysis of variance revealed no significant differences between responders and non-responders either at the end of therapy (*p* = 0.28) or at the end of the observational range (*p* = 0.27). Some variability in score changes was noted: non-responders displayed a gradual buildup of positive effects toward the end of treatment, whereas responders sustained these benefits during the subsequent observation period.

An assessment of the clinical efficacy of Taxifolin Aqua revealed a statistically significant positive effect on cognitive function. An analysis of variance (ANOVA) explored changes in the composite cognitive scores (MMSE, MoCA) 4 weeks after the therapy concluded. The results showed significant differences between the responders and non-responders (F(2,20) = 17.38, *p* = 4.22e-05). By the end of the 8-week treatment, patients showed significant clinical improvement, which continued for most of the following month.

During the study period of Taxifolin Aqua, no adverse events were identified in patients with aMCI.

Among the biomarkers studied (8, 9), only two showed significant changes during treatment in the total group compared with baseline, as adjusted for multiple comparison. These were the ratio of alpha-1PI to NE activities (*p* = 0.034) and platelet SOD concentration (*p* = 0.023). The platelet GR activity displayed a complex interplay between clinical response and the parameter’s dynamics. For non-responders, the Friedman test revealed significant changes between visits (*p* = 0.004). However, in responders, no changes were noted (*p* = 0.913). In responders, the change in platelet GR levels at the end of treatment was significantly different from that in non-responders (*p* = 0.022). This observation suggests that platelet GR dynamics may reflect different adaptive mechanisms in patients with different types of response to therapy. Analyzing the links between biomarker changes and clinical outcomes uncovered a notable correlation: the change in platelet GR levels by the end of treatment showed a significant negative correlation with the patient’s clinical response (R = −0.547, *p* = 0.007). When analyzing individual predictors of response, baseline CPK, GR, GPx, and GDH levels significantly differed between responders and non-responders (*p* < 0.05), suggesting their potential prognostic value.

## Limitations

The open-label design of our study introduces potential bias. Because of open-label design, the researchers could not include any aMCI group treated with placebo. Thus, a limited control group of patients with aMCI, whose cognitive abilities matched those of the Taxifolin-treated sample, was monitored by the same team of psychiatrists. The authors recognize that, and future studies should consider a double-blind, placebo-controlled approach. In our pilot study, the sample size is rather small, which limits statistical power and generalizability. Thus, the results can be regarded as preliminary and requiring confirmation on expanded groups. The results on biomarkers can also be categorized as exploratory. This is due to the lack of data on these biomarkers in the control group.

## Conclusion

Our study has its constraints, but we believe that the 48% responders’ portion and the 2.73-point clinically meaningful improvement on the MoCA score are compelling reasons to continue exploring Taxifolin Aqua through larger, controlled trials with MCI patients. Statistically significant effects on individual biological parameters, in particular the alpha-1PI/NE, GR, and platelet SOD, may indicate potential mechanisms of the medicine action associated with modulation of oxidative stress and anti-inflammatory activity.

## Data Availability

The raw data supporting the conclusions of this article will be made available by the authors, without undue reservation.

## Supplementary material

Supplementary Figure 1 Dynamics of MoCA scores in patients with aMCI treated with Taxifolin Aqua (green and red for responders or non-responders, respectively), or Galavit (comparison group, “Control” blue). Means are given bold for all three groups.

Supplementary Table 1 Stratification of patients by response groups (composite classification method).

Supplementary Table 2 Demographic and clinical characteristics of responders and non-responders. ^1^Between-group comparison of responders and non-responders; ^2^Wilcoxon test for independent samples; ^3^Pearson chi-square test statistics; SD, Standard Deviation; Q1/Q3, quartiles 1/3; Min, Max, minimum, maximum value.
