# Supplementary material for: Pilot clinical study of Taxifolin Aqua effect in patients with amnestic mild cognitive impairment
Source: Front Psychiatry. 2026 Sep 28;17:1814042. doi: 10.3389/fpsyt.2026.1814042 (PMC13655543; doi:10.3389/fpsyt.2026.1814042)
Supplement: Supplementary file 1 [file Table1.docx]

Table 1 Supplement. Stratification of patients by response groups (composite classification method)

| Visit | Total observation number | Responder | Non-responder | Portion of responders, % |
| --- | --- | --- | --- | --- |
| V3 | 23 | 16 | 7 | 69.7 |
| V4 | 23 | 15 | 8 | 65.2 |
