# Supplementary material for: Pilot clinical study of Taxifolin Aqua effect in patients with amnestic mild cognitive impairment
Source: Front Psychiatry. 2026 Sep 28;17:1814042. doi: 10.3389/fpsyt.2026.1814042 (PMC13655543; doi:10.3389/fpsyt.2026.1814042)
Supplement: Supplementary file 2 [file Table2.docx]

Table 2. Demographic and Clinical Characteristics of Responders and Non-Responders

| Demographic data | Total group N = 23 | Clinical Response Groups | | *p*-value^1^ |
| --- | --- | --- | --- | --- |
|  |  | Non-responders N = 12 | Responders N = 11 |  |
| Age, years |  |  |  | 0.131^2^ |
| Mean ± SD | 67.70 ± 9.04 | 70.50 ± 9.55 | 64.64 ± 7.72 |  |
| Median [Q1, Q3] | 68.00  [61.00, 74.00] | 69.50  [64.50, 75.00] | 66.00  [59.00, 69.00] |  |
| Min, Max | 53.00, 87.00 | 53.00, 87.00 | 53.00, 78.00 |  |
| Gender, n (%) |  |  |  | >0.999^3^ |
| Female | 18 (78%) | 9 (75%) | 9 (82%) |  |
| Male | 5 (22%) | 3 (25%) | 2 (18%) |  |
| Age of manifestation, years |  |  |  | 0.048^2^ |
| Mean ± SD | 62.39 ± 7.55 | 65.25 ± 7.14 | 59.27 ± 7.00 |  |
| Median [Q1, Q3] | 63.00  [58.00, 69.00] | 66.00  [61.50, 70.50] | 60.00  [53.00, 64.00] |  |
| Min, Max | 48.00, 75.00 | 50.00, 75.00 | 48.00, 72.00 |  |
| Brain atrophy according to MRI, degree, n (%) |  |  |  | 0.128^3^ |
| Normal/Very Mild | 14 (61%) | 5 (42%) | 9 (82%) |  |
| Mild to Moderate | 8 (35%) | 6 (50%) | 2 (18%) |  |
| Moderately Severe to Severe | 1 (4.3%) | 1 (8.3%) | 0 (0%) |  |
| White matter foci according to MRI, degree, n (%) |  |  |  | 0.977^3^ |
| No foci | 8 (35%) | 4 (33%) | 4 (36%) |  |
| Small, single foci | 11 (48%) | 6 (50%) | 5 (45%) |  |
| Small, multiple foci | 4 (17%) | 2 (17%) | 2 (18%) |  |
| Modified Hachinski ischaemic scale, score, n (%) |  |  |  | 0.008^3^ |
| 0 | 7 (30%) | 0 (0%) | 7 (64%) |  |
| 1 | 1 (4.3%) | 0 (0%) | 1 (9.1%) |  |
| 2 | 13 (57%) | 10 (83%) | 3 (27%) |  |
| 3 | 1 (4.3%) | 1 (8.3%) | 0 (0%) |  |
| 4 | 1 (4.3%) | 1 (8.3%) | 0 (0%) |  |

Notes: ^1^ – Between-group comparison of responders and non-responders; ^2^ – Wilcoxon test for independent samples; ^3^ – Pearson chi-square test statistics; SD – Standard Deviation; Q1/Q3 – quartiles 1/3; Min, Max – minimum, maximum value.
